# Mutations in *HISTONE ACETYLTRANSFERASE1* affect sugar response and gene expression in Arabidopsis

**DOI:** 10.3389/fpls.2013.00245

**Published:** 2013-07-17

**Authors:** Timothy J. Heisel, Chun Yao Li, Katia M. Grey, Susan I. Gibson

**Affiliations:** Department of Plant Biology, Microbial and Plant Genomics Institute, University of MinnesotaSaint Paul, MN, USA

**Keywords:** histone acetyltransferase, chromatin modification, SnRK1, sugar signaling, sugar response, Arabidopsis, fertility, sucrose response

## Abstract

Nutrient response networks are likely to have been among the first response networks to evolve, as the ability to sense and respond to the levels of available nutrients is critical for all organisms. Although several forward genetic screens have been successful in identifying components of plant sugar-response networks, many components remain to be identified. Toward this end, a reverse genetic screen was conducted in *Arabidopsis thaliana* to identify additional components of sugar-response networks. This screen was based on the rationale that some of the genes involved in sugar-response networks are likely to be themselves sugar regulated at the steady-state mRNA level and to encode proteins with activities commonly associated with response networks. This rationale was validated by the identification of *hac1* mutants that are defective in sugar response. *HAC1* encodes a histone acetyltransferase. Histone acetyltransferases increase transcription of specific genes by acetylating histones associated with those genes. Mutations in *HAC1* also cause reduced fertility, a moderate degree of resistance to paclobutrazol and altered transcript levels of specific genes. Previous research has shown that *hac1* mutants exhibit delayed flowering. The sugar-response and fertility defects of *hac1* mutants may be partially explained by decreased expression of AtPV42a and AtPV42b, which are putative components of plant SnRK1 complexes. SnRK1 complexes have been shown to function as central regulators of plant nutrient and energy status. Involvement of a histone acetyltransferase in sugar response provides a possible mechanism whereby nutritional status could exert long-term effects on plant development and metabolism.

## Introduction

The ability to sense and respond to nutrients, such as soluble sugars, is shared by all living organisms. In fact, metabolic response networks are likely to have been among the first response networks to evolve. Nutrient response networks play fundamental roles in regulation of gene expression and diverse metabolic and developmental processes. In plants, the levels of soluble sugars, such as glucose (Glc) and sucrose (Suc), affect developmental processes ranging from seed, embryo, and meristem development (Borisjuk et al., 2003, 2004; Radchuk et al., 2010; Eveland and Jackson, 2012), to seed germination (Pego et al., 1999; Finkelstein and Lynch, 2000; To et al., 2002; Ullah et al., 2002; Price et al., 2003; Dekkers et al., 2004; Li et al., 2012), root:shoot ratios (Wilson, 1988), induction of flowering (King and Evans, 1991; Bernier et al., 1993; Corbesier et al., 1998; Roldán et al., 1999; van Dijken et al., 2004; Funck et al., 2012; Wahl et al., 2013), formation of adventitious roots (Takahashi et al., 2003), senescence (Quirino et al., 2000; Paul and Pellny, 2003; Wingler et al., 2009; Thomas, 2013), and overall plant growth (reviewed in Smeekens et al., 2010).

Studies on the molecular mechanisms underlying sugar responses indicate that plants utilize several sugar-response pathways (Chiou and Bush, 1998; Loreti et al., 2000; Xiao et al., 2000; Tiessen et al., 2003; Rolland et al., 2006; Smeekens et al., 2010; Eveland and Jackson, 2012). A few components of plant sugar-response pathways have been identified by characterizing plant homologs of genes shown to act in sugar response in other organisms. For example, SUCROSE NON-FERMENTING1 (SNF1) and AMP-ACTIVATED KINASE (AMPK) play critical roles in metabolite response in yeast and animals, respectively. The plant homologs of these genes have been designated as SNF1-RELATED PROTEIN KINASE1s (SnRK1s, reviewed in Ghillebert et al., 2011). Characterization of SnRK1 complexes revealed that they play a central role in regulating energy homeostasis and sugar signaling in plants (Halford and Hardie, 1998; Halford et al., 2003; Baena-González et al., 2007; Lu et al., 2007; Polge and Thomas, 2007; Halford and Hey, 2009; Jossier et al., 2009; Radchuk et al., 2010; Delatte et al., 2011). HEXOKINASE2 (HXK2) and several proteins involved in G protein signaling have also been shown to play important roles in sugar response in yeast (reviewed in Rolland et al., 2002b). HEXOKINASE1 (HXK1) has been shown to play an important role in sugar response by acting as a Glc sensor in plants. Plants carrying mutations in *HXK1* exhibit multiple defects in sugar response, including reduced sensitivity to the inhibitory effects of high sugar concentrations on early seedling development and alterations in Glc-regulated expression of specific genes (Jang et al., 1997; Moore et al., 2003; Cho et al., 2006; Karve et al., 2012; Granot et al., 2013). REGULATOR OF G PROTEIN SIGNALING1 (RGS1) and G-PROTEIN ALPHA SUBUNIT1 (GPA1) act together as a Glc sensor in plants (Chen et al., 2003, 2006; Chen and Jones, 2004; Grigston et al., 2008).

Components of plant sugar-response networks have also been identified via forward genetic screens. Early seedling development of Arabidopsis is inhibited by exposure to high concentrations of exogenous Glc or Suc during approximately the first 40 h after the start of imbibition. Wild-type seeds sown on media supplemented with 0.3 M Glc or Suc germinate, but only a small percentage of wild-type seeds are able to develop into seedlings with expanded cotyledons and true leaves (Gibson et al., 2001). This sugar-mediated developmental arrest has been used as the basis for several forward genetic screens. Interestingly, characterization of some of the mutants identified via these screens has revealed the existence of significant crosstalk between sugar-response pathways and a number of pathways involved in phytohormone biosynthesis and response (Zhou et al., 1998; Arenas-Huertero et al., 2000; Laby et al., 2000; Gibson et al., 2001; Huang et al., 2008). Forward genetic screens for mutants defective in sugar-regulated expression of specific genes has also resulted in identification of components of plant sugar response pathways (Dijkwel et al., 1997; Martin et al., 1997; Mita et al., 1997a,b; Rook et al., 2001).

Although forward genetic screens and homology-based approaches have resulted in the identification of a number of components of plant sugar-response networks, many more components remain to be identified. In this work we report the development of a reverse genetic screen to identify additional components of plant sugar-response networks. Using this screen, mutations in *HISTONE ACETYLTRANSFERASE1* (*HAC1*) were determined to cause a sugar-response defect in Arabidopsis. Histone modifying proteins help to control gene expression. Acetylation in particular is a reversible modification that cells use to control the accessibility of specific genes to polymerases (Struhl, 1998). Hyperacetylation is linked with increased gene expression and deacetylation is linked with decreased gene expression (Hassig and Schreiber, 1997; Struhl, 1998; Ahringer, 2000). Histone acetyltransferase families include the p300/CBP family, the GNAT-MYST family and the TAF_*II*_250 family (Pandey et al., 2002). HAC1 is one of five related CBP/p300-like histone acetyltransferases in Arabidopsis (Pandey et al., 2002). In addition to acetylating histones, members of the p300/CBP family of histone acetyltransferases acetylate a number of non-histone proteins, including transcription factors, nuclear receptor co-activators, and hormone receptors (Sterner and Berger, 2000; Glozak et al., 2005; Kimura et al., 2005). HAC1 has been demonstrated to be actively involved both in histone acetylation as well as in protein-protein binding with transcription factors and other transcriptional activators (Bordoli et al., 2001; Pandey et al., 2002; Bharti et al., 2004). Mutations in *HAC1* have been shown to cause delayed flowering times in Arabidopsis by indirectly increasing the expression of the central floral repressor, FLC (Deng et al., 2007; Han et al., 2007). Mutations in *HAC1* have also been shown to cause decreased fertility in Arabidopsis (Deng et al., 2007; Han et al., 2007) and to reduce the rates of Agrobacterium-mediated root transformation (Crane and Gelvin, 2007). Results presented here demonstrate that mutations in *HAC1* cause a sugar-response defect and lead to decreased expression of specific genes, including putative components of SnRK1 complexes.

## Results

### Selection of target genes

Several different forward genetic screens have been used successfully to identify mutants with altered response to the levels of soluble sugars, such as Glc, Suc, and mannose. However, forward genetic screens may not allow identification of all the genes involved in sugar response. In cases where two or more genes carry out redundant functions, mutating just one of those genes may cause only a weak phenotype that is difficult to detect via a forward genetics approach. Use of both reverse and forward genetics approaches may thus allow identification of a greater number of the genes that affect a given process. Toward this end, a reverse genetics approach was used to identify genes involved in sugar response.

The reverse genetics approach used in this study was based on the rationale that some of the genes involved in sugar response might themselves be regulated by sugars at the steady-state mRNA level. It should be noted that the success of this strategy is not dependent on all of the genes involved in sugar response being sugar regulated. In theory, a reverse genetic screen based on this rationale may be successful if even only one gene involved in sugar response is itself sugar regulated. To further narrow the list of candidate genes emphasis was placed on genes predicted to encode proteins with activities commonly associated with response networks. Examples of such activities include transcriptional regulation, protein phosphorylation and dephosphorylation, and chromatin modification. Consequently, genes that are sugar regulated at the steady-state mRNA level and that are predicted to encode proteins with activities commonly associated with response networks were considered to be candidates to act in a sugar-response network.

Sugar-regulated genes were identified by incubating *Arabidopsis thaliana* seeds on minimal media for 20 h, followed by an additional 12–13 h incubation on minimal media supplemented with 0.1 M Glc, Suc, or sorbitol (as an osmotic control). This time course was chosen based on findings that wild-type seedlings are only sensitive to the inhibitory effects of high concentrations of exogenous Glc or Suc on early seedling development during the first 32–48 h after the start of imbibition (Gibson et al., 2001). RNA samples extracted from harvested tissues were analyzed using Affymetrix ATH1 GeneChips, which contain primer sets for approximately 24,000 Arabidopsis genes (Redman et al., 2004). Approximately 1100 genes that exhibit significant alterations in expression levels on Glc and/or Suc compared to equi-molar sorbitol were identified (Pattison et al., unpublished results). This list was narrowed down to 189 target genes. In most cases these target genes are predicted to code for proteins commonly found in response networks, such as transcription factors, protein phosphatases, protein kinases, and chromatin modifying proteins.

### Identification and characterization of lines carrying mutations in target genes

Results indicating that a gene is sugar regulated and is predicted to encode a protein with a function commonly associated with response networks are not sufficient to determine whether that gene actually acts in sugar response. To test whether any of the 189 target genes function in sugar response, T-DNA insertion mutants were identified for as many of the target genes as possible. Putative T-DNA insertion mutants were obtained from the Arabidopsis Biological Resource Center for 170 of the 189 genes. Most of these mutants were generated as part of the SALK collection (Alonso et al., 2003) and the remaining mutants were generated as part of the SAIL collection (Sessions et al., 2002). Multiple independent mutant lines were obtained for some target genes, so that a total of over 200 unique T-DNA lines were obtained. These lines were screened by PCR for the presence of T-DNA inserts in predicted locations, as described (Alonso et al., 2003). Seedlings from lines confirmed to carry the predicted T-DNA inserts were then screened to identify plants that were homozygous for the T-DNA insertions. These efforts resulted in the identification of plants homozygous for mutations in approximately 130 of the 189 target genes.

Seeds from 109 of the homozygous mutant lines were screened for defects in sugar response. High (e.g., 0.3 M) concentrations of exogenous Suc or Glc have been shown to inhibit early seedling development. Seeds sown on media containing high concentrations of Suc or Glc germinate, but the vast majority fail to develop into seedlings with true leaves and expanded cotyledons. Instead, the majority of the seedlings arrest development. These high concentrations of exogenous sugars certainly cause osmotic stress. However, the effects of high concentrations of Glc or Suc on early seedling development cannot be solely explained by osmotic stress as equi-molar concentrations of sorbitol do not exert the same effects (Laby et al., 2000). Mutant lines were tested for a sugar-insensitive phenotype by sowing 50–100 seeds from each line on media containing approximately 0.3 M Glc or Suc. Mutant lines were also tested for a sugar-hypersensitive phenotype by sowing on media supplemented with 0.22–0.23 M Glc or Suc.

### Identification of sugar-response mutants

The screen for sugar-hypersensitive mutants resulted in the identification of a line carrying a T-DNA insert in AT5G51760. AT5G51760 encodes a protein phosphatase 2 c designated as *ABA-HYPERSENSITIVE GERMINATION1* (*AHG1*). Mutations in *AHG1* have been shown previously to cause abscisic acid, Glc, Suc, and mannitol hypersensitive seed germination phenotypes (Nishimura et al., 2007). The screen for sugar-insensitive mutants resulted in the identification of a line that exhibits resistance to the inhibitory effects of both exogenous Glc and Suc on early seedling development. This line carries a mutation in AT1G79000. AT1G79000 encodes *HISTONE ACETYLTRANSFERASE1* (*HAC1*). Further efforts focused on characterization of *HAC1* as mutations in *HAC1* had not previously been shown to affect sugar response.

**Figure 1:**
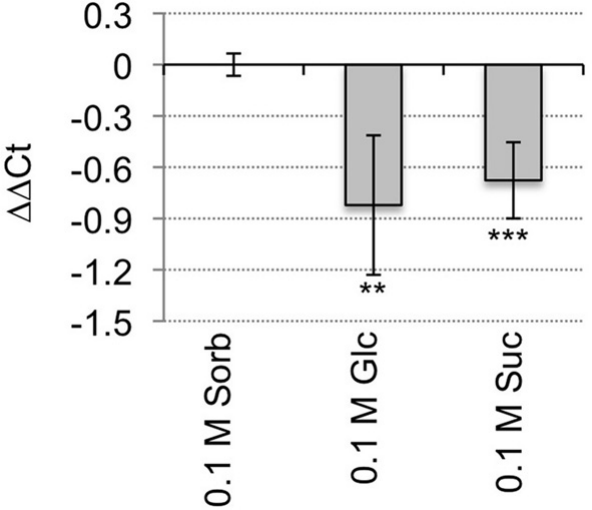
***HAC1* expression is repressed by sugars**. Wild-type Arabidopsis seeds were surface sterilized and then incubated in the dark at 4°C for 3 days prior to being sown on nytex screens on Petri plates containing solid minimal Arabidopsis media. The seeds were incubated under continuous light at room temperature for 20 h, and then the nytex screens and seeds were transferred to Petri plates containing Arabidopsis minimal media supplemented with 0.1 M sorbitol (Sorb), Glc, or Suc. After an additional 12–13 h, seeds were harvested, followed by isolation of RNA. The RNA was then used for qRT-PCR analysis. The transcript levels of *ACT7* and *UBQ6* were determined and the geometric means of their Ct used to normalize transcript levels. Transcript levels are expressed as ΔΔCt. ΔΔCt = ΔCt_*HAC1*_ on sorbitol-Δ Ct_*HAC1*_ on indicated media. ΔCt = Ct_*HAC1*_ on indicated media-Ct_*ACT7/UBQ6*_ on same media. Negative ΔΔCt values indicate that *HAC1* transcript levels are lower in wild-type seeds germinating on the indicated media than in wild-type seeds germinating on sorbitol. Six technical replicates were performed for each biological replicate. Error bars indicate standard deviations. *HAC1* expression on sorbitol vs. Glc or Suc differed with: ^**^p < 0.05; or ^***^p < 0.02, according to a Student's *t*-test. *N* = 3.

**Figure 2:**
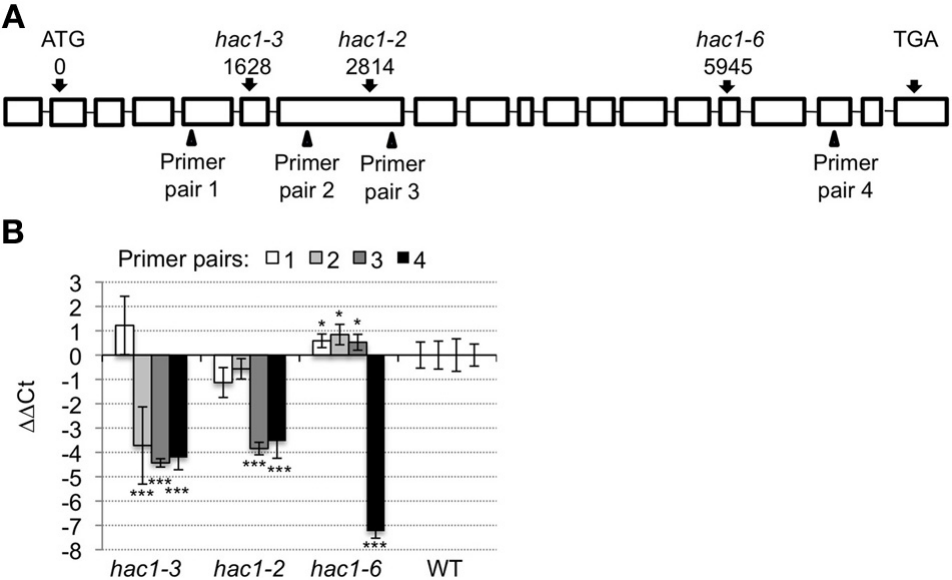
***HAC1* T-DNA locations and affects on *HAC1* expression. (A)** Boxes indicate exons and horizontal lines indicate introns in the *HAC1*. The locations of the T-DNA inserts in the *HAC1* genomic sequence (AT1G79000.2) are indicated with respect to the first nucleotide of the start codon. The positions of the four primer pairs used for the qRT-PCR analyses depicted in panel (B) are indicated by triangles. **(B)** Mutant and wild-type (WT) Arabidopsis seeds were surface sterilized and then incubated in the dark at 4°C for 3 days prior to being sown on nytex screens on Petri plates containing solid minimal Arabidopsis media. The seeds were incubated under continuous light at room temperature for 20 h, and then the nytex screens and seeds were transferred to Petri plates containing Arabidopsis minimal media supplemented with 0.1 M sorbitol. After an additional 12–13 h, seeds were harvested, followed by isolation of RNA. Quantitative RT-PCR was then used to measure transcript levels. The transcript levels of *ACT7* and *UBQ6* were determined and the geometric means of their Ct used to normalize transcript levels. Transcript levels are expressed as ΔΔCt. ΔΔCt = ΔCt_indicated pimer pair in wild-type_ – ΔCt_indicated primer pair in indicated line_. ΔCt = Ct_indicated primer pair in indicated line_ – Ct_*ACT7*/*UBQ6*_ in same line. Positive ΔΔCt values indicate that *HAC1* transcript levels appear higher in the indicated *hac1* line than in wild-type when measured using the indicated primer pair. Conversely, negative ΔΔCt values indicate that *ACT7* transcript levels appear lower in the indicated *hac1* line than in wild-type when measured using the indicated primer pair. Two technical replicates were performed for each biological replicate. Error bars indicate standard deviations. For each primer pair, the Student's *t*-test was used to compare *HAC1* transcript levels in each *hac1* mutant line vs. wild-type. ^*^p < 0.1; or ^***^p < 0.02, according to a Student's *t*-test. *N* = 4.

*HAC1* is predicted to encode a histone acetyltransferase that is orthologous to the p300/CREB-binding protein gene family in mammalian and yeast systems (Bordoli et al., 2001; Pandey et al., 2002). *HAC1* steady-state mRNA levels are significantly lower in wild-type seeds germinating on media supplemented with Suc or Glc than in wild-type seeds germinating on media supplemented with an equi-molar concentration of sorbitol (Figure 1). To test further whether mutations in *HAC1* are responsible for the sugar-response phenotype, additional lines predicted to carry T-DNA insertions in *HAC1* were obtained from the Arabidopsis Biological Resource Center. Homozygous mutants were identified for a total of three independent lines that carry T-DNA inserts in *HAC1*. The precise locations of the T-DNA inserts in these lines were determined by PCR amplification of DNA flanking the T-DNA insertion sites, followed by DNA sequencing of the PCR products (Figure 2A). Quantitative RT-PCR was used to determine the effects of the T-DNA insertions on *HAC1* transcript levels (Figure 2B). For each *hac1* mutant tested, PCR primers located 5′ of the T-DNA insertion site in that *hac1* line detected approximately wild-type *HAC1* transcript levels. In contrast, PCR primers located 3′ of T-DNA insertion sites detected greatly reduced *HAC1* transcript levels in *hac1* mutants as compared to wild-type plants. These results indicate that all three *hac1* mutants produce partial *HAC1* transcripts that are similar in abundance to the full-length *HAC1* transcripts produced by wild-type plants.

### *hac1* mutants are resistant to the inhibitory effects of high sugar concentrations

**Figure 3:**
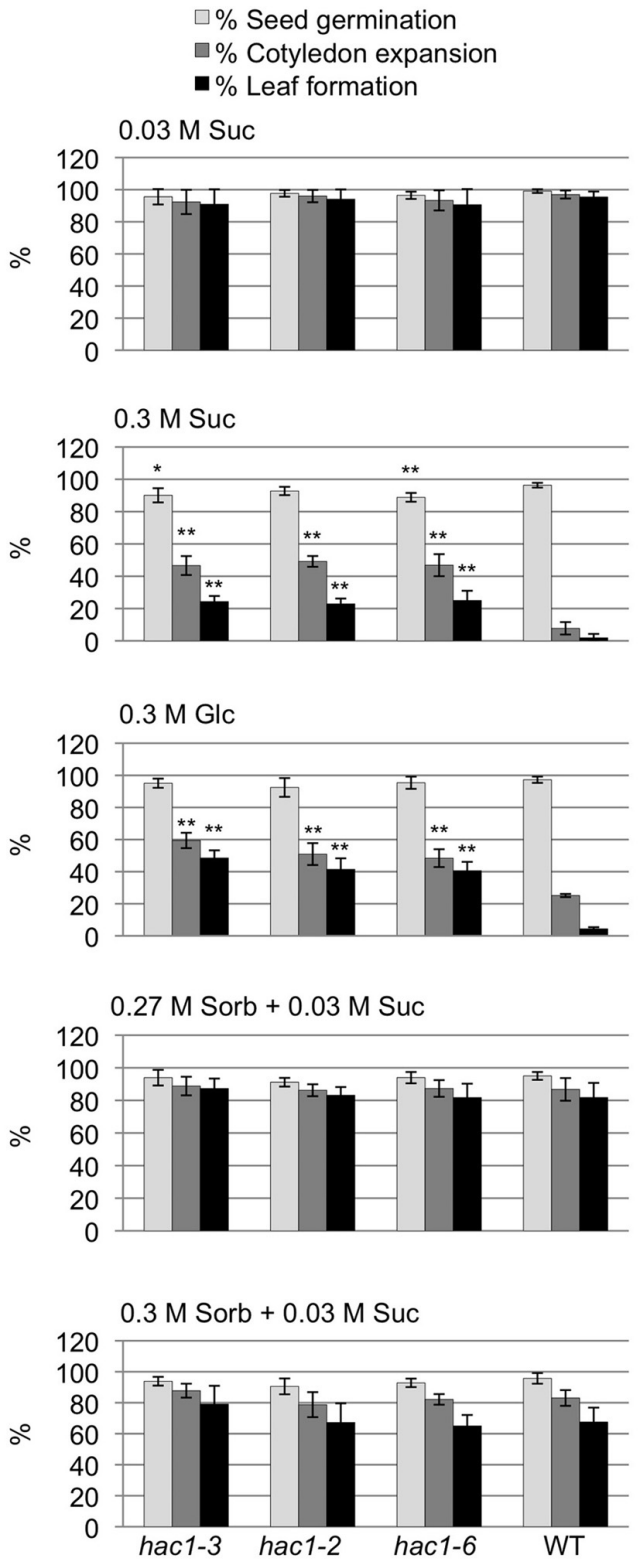
**The *hac1* mutants are resistant to the inhibitory effects of high concentrations of exogenous sugars on early seedling development**. Mutant and wild-type Col-0 (WT) seeds were surface sterilized and then incubated in the dark at 4°C for 3 days prior to being sown on solid Arabidopsis minimal media supplemented with the indicated additive. After an additional 14 days in the light at room temperature, seeds were scored to determine the percentages of seeds that had germinated, formed expanded cotyledons and developed true leaves. Error bars indicate standard deviations. Mutant and wild-type results differed with: ^*^p < 0.05; or ^**^p < 0.01, according to a Student's *t*-test. *N* = 4.

Characterization of the *hac1* lines revealed that all three lines tested are resistant to the inhibitory effects of high concentrations of exogenous Glc and Suc on early seedling development (Figure 3). Unlike wild-type seeds, a significant percentage of *hac1* seeds sown on media containing 0.3 M Glc or Suc are able to develop into seedlings with true leaves and expanded cotyledons. The sugar-resistant phenotype of the *hac1* mutants cannot be explained solely by an osmo-tolerant phenotype. Mutant and wild-type seeds exhibit similar degrees of sensitivity to sorbitol concentrations that are equi-molar, or slightly higher than equi-molar, to those used to examine Glc and Suc sensitivity (Figure 3).

### *hac1* mutants are moderately resistant to paclobutrazol

**Figure 4:**
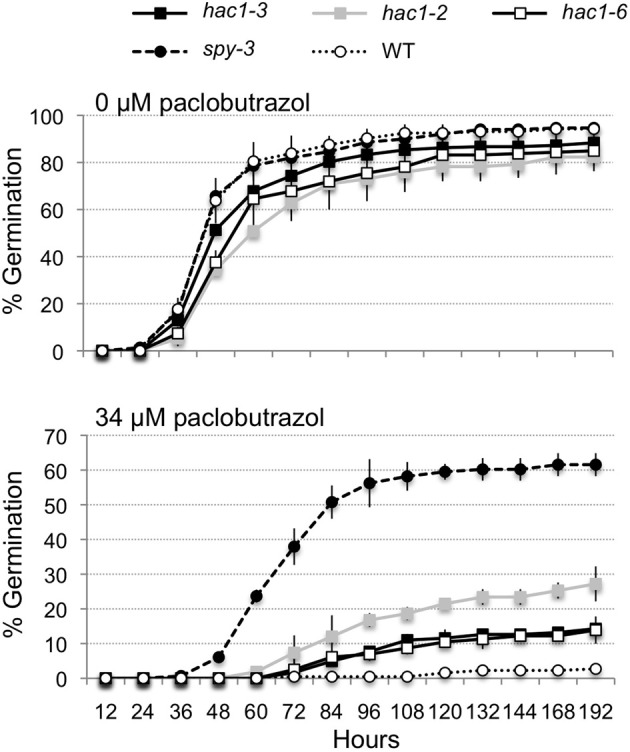
**The *hac1* mutants are moderately resistant to the inhibitory effects of paclobutrazol on seed germination**. Mutant and wild-type Col-0 (WT) seeds were surface sterilized, imbibed in the dark at 4°C for 3 days and then sown on Petri plates with Arabidopsis minimal media and 0 or 34 μM paclobutrazol. The Petri plates were incubated under continuous light at 22°C and scored for seed germination at regular intervals. The *spy-3* mutant (Jacobsen and Olszewski, 1993) was included as a positive control. Error bars indicate standard deviations. *N* = 2.

Mutations that lead to sugar-insensitive or hypersensitive phenotypes often also affect phytohormone response. For example, a number of sugar-insensitive mutants have been found to be resistant to the inhibitory effects of abscisic acid and/or paclobutrazol (an inhibitor of the biosynthesis of gibberellins) on seed germination (Arenas-Huertero et al., 2000; Huijser et al., 2000; Laby et al., 2000; Gibson et al., 2001; Rook et al., 2001; Huang et al., 2008). Therefore, *hac1* mutants were tested for altered sensitivity to the inhibitory effects of paclobutrazol on seed germination. All three of the *hac1* mutants tested exhibited slightly faster rates of seed germination on media supplemented with 34 μM paclobutrazol than did seeds from wild-type plants (Figure 4). In addition, the percentages of seeds that germinated within 8 days on 34 μM paclobutrazol were significantly higher for each of the three *hac1* mutant lines tested than for the wild-type line. Comparison of the percentages of *hac1* and wild-type seeds that had germinated within 8 days on 34 μM paclobutrazol showed that these percentages differed with *p* values of 0.02, 0.0002, and 0.06 for *hac1-2, hac1-3*, and *hac1-6*, respectively. However, the germination rates of the *hac1* mutants were much lower on 34 μM paclobutrazol than the germination rates of the *spy-3* mutant. The *spy-3* mutant was previously identified as a paclobutrazol-resistant mutant (Jacobsen and Olszewski, 1993) and was included in these experiments as a positive control. Thus, the results of these experiments indicate that mutations in *HAC1* confer a modest degree of resistance to the inhibitory effects of paclobutrazol on seed germination.

### *hac1* mutants are defective in seed production

**Figure 5:**
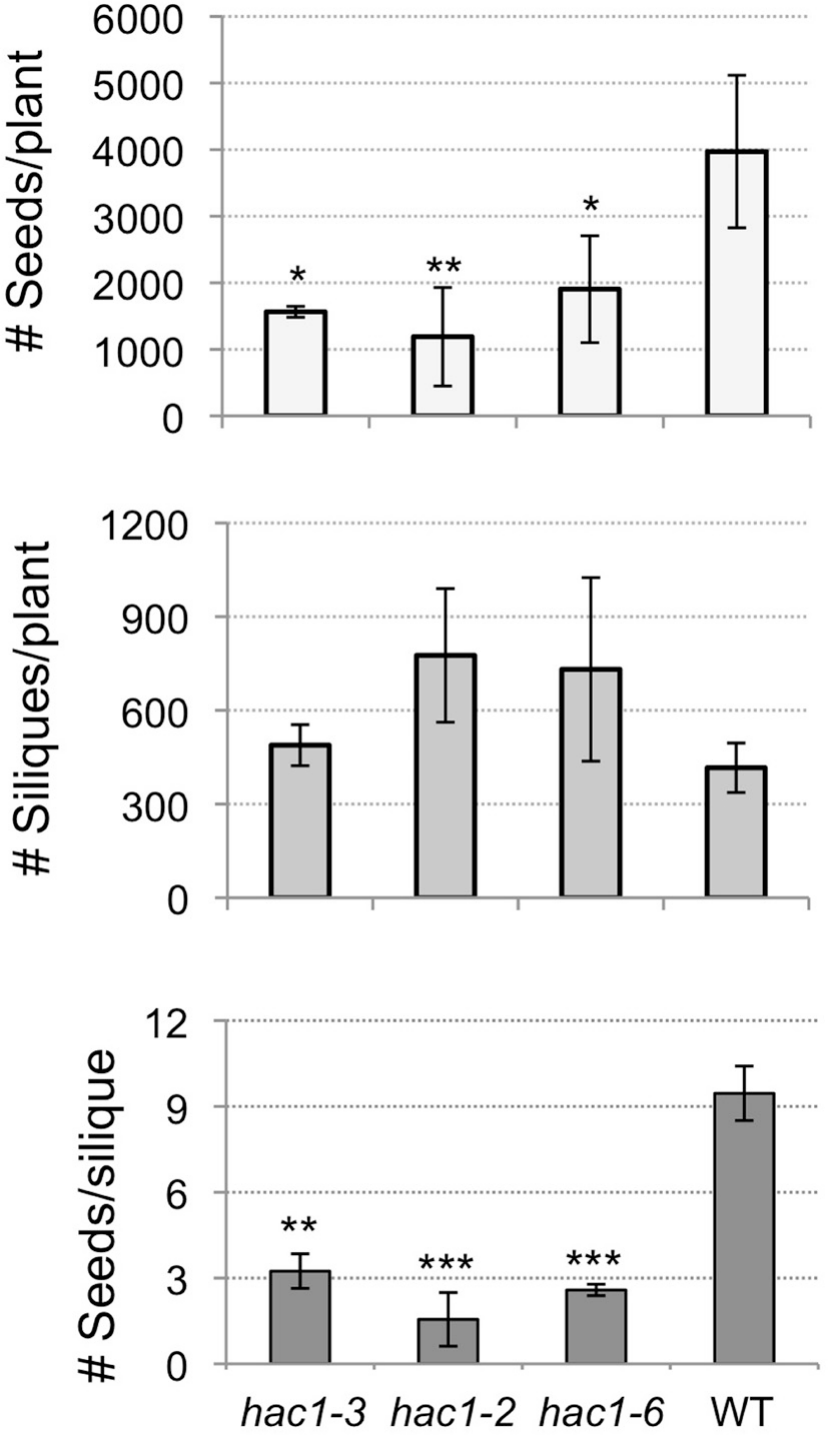
**Mutations in *HAC1* reduce seed production**. Mutant and wild-type plants were grown to maturity. The numbers of siliques on each plant were counted. Siliques were then collected from each plant and the total number of seeds produced per silique and per plant determined. Error bars indicate standard deviations. Mutant and wild-type results differed with: ^*^p < 0.1; ^**^p < 0.05; or ^***^p < 0.01, according to a Student's *t*-test. *N* = 2 (*hac1-3*, wild-type, WT) or 3 (*hac1-2, hac1-6*).

Adult *hac1* plants were observed to produce many siliques that were extremely stunted and which contained few or no seeds. To characterize this phenotype the numbers of siliques produced per plant, seeds produced per plant, and seeds produced per silique were determined for *hac1* and wild-type plants. Although the average number of siliques produced by *hac1* plants was greater than the average number produced by wild-type plants, these differences in silique number were not statistically significant between the samples analyzed (Figure 5). However, analysis of the number of seeds produced per plant revealed that *hac1* plants produce significantly fewer seeds than do wild-type plants (Figure 5). As the reduced seed production of *hac1* plants does not appear to be due to a reduction in the number of siliques per plant, the numbers of seeds per silique were analyzed. Siliques produced by *hac1* plants were found to contain significantly fewer seeds on average than siliques produced by wild-type plants (Figure 5). Thus, the reduced seed production of *hac1* plants appears to be due to a decrease in the number of seeds per silique, rather than in the number of siliques per plant.

### Mutations in *hac1* affect the expression of specific genes

Histone acetyltransferases cause increased transcription of specific genes by modifying histones associated with those genes. Mutations in *HAC1* were thus anticipated to affect the transcription of specific genes. Preliminary experiments were conducted using Affymetrix Arabidopsis ATH1 GeneChips to identify genes that might be expressed at altered levels in *hac1* compared to wild-type germinating seeds (data not shown). Quantitative RT-PCR was then used to analyze the expression of some of the genes that appeared most likely to be expressed at altered levels in *hac1* mutants. In addition, five genes that were previously shown to be sugar-regulated (AT2G28900, AT3G08940, AT4G35770, AT5G07990, and AT5G13930) were included in these experiments.

**Figure 6:**
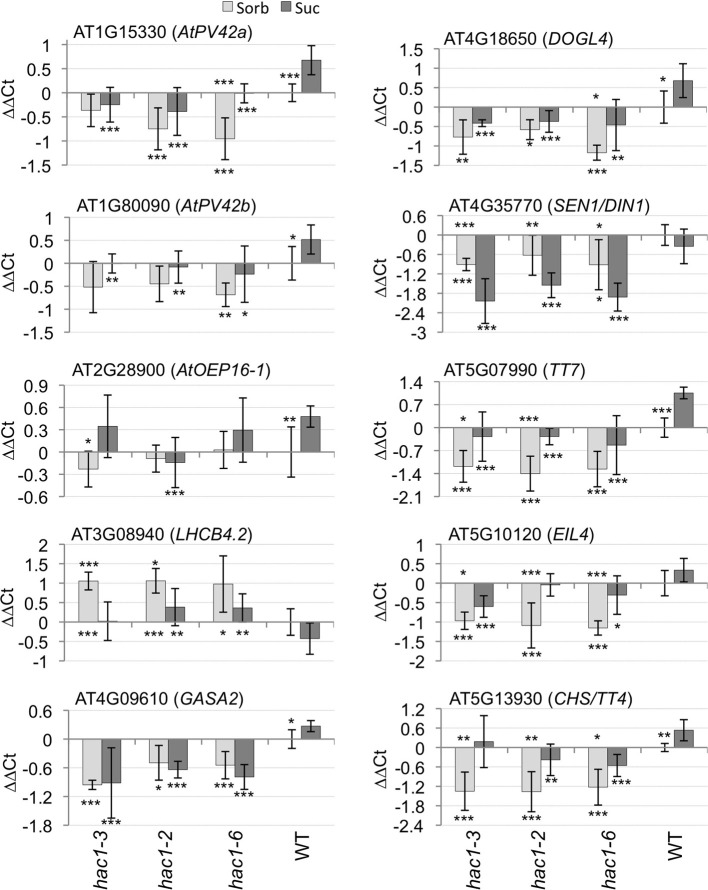
**Mutations in *HAC1* cause altered expression of specific genes**. Mutant and wild-type (WT) Arabidopsis seeds were surface sterilized and then incubated in the dark at 4°C for 3 days prior to being sown on nytex screens on Petri plates containing solid minimal Arabidopsis media. The seeds were incubated under continuous light at room temperature for 20 h, and then the nytex screens and seeds were transferred to Petri plates containing Arabidopsis minimal media supplemented with 0.1 M sorbitol (Sorb) or Suc. After an additional 12–13 h, seeds were harvested, followed by isolation of RNA. Quantitative RT-PCR was then used to measure transcript levels. The transcript levels of *ACT7* and *UBQ6* were determined and the geometric means of their Ct used to normalize transcript levels. Transcript levels are expressed as ΔΔCt. ΔΔCt = ΔCt_indicated gene in wild-type on sorbitol_ – ΔCt_indicated gene in indicated line on indicated media_. ΔCt = Ct_indicated gene in indicated line on indicated media_ – Ct_*ACT7/UBQ6*_ in same line on same media. Positive ΔΔCt values indicate that transcript levels for the indicated gene are higher in the indicated line on the indicated media than in wild-type seeds incubated on sorbitol. Conversely, negative ΔΔCt values indicate that transcript levels for the indicated gene are lower in the indicated line on the indicated media than in wild-type seeds incubated on sorbitol. Two technical replicates were performed for each biological replicate. Error bars indicate standard deviations. The results of Student's *t*-tests comparing transcript levels for seeds germinated on 0.1 M sorbitol vs. seeds of the same line germinated on 0.1 M Suc are indicated by asterisks above the columns indicating the average transcript levels for seeds germinated on 0.1 M sorbitol. The results of Student's t-tests comparing transcript levels in *hac1* vs. wild-type seeds germinating on the same media are indicated by asterisks below the columns indicating the average transcript levels for each *hac1* allele on each media. ^*^p < 0.1; ^**^p < 0.05; or ^***^p < 0.02, according to a Student's *t*-test. *N* = 4.

Nine of the genes analyzed by quantitative RT-PCR exhibit consistent alterations in expression in *hac1* vs. wild-type germinating seeds (Figure 6). Eight of these genes exhibit significantly lower transcript levels in *hac1* than in wild-type germinating seeds. The ninth gene (AT3G08940) is expressed at significantly higher levels in *hac1* than in wild-type germinating seeds. The tenth gene analyzed (AT2G28900) does not exhibit consistently different *HAC1* transcript levels in *hac1* vs. wild-type. AT1G15330 and AT1G80090 encode AtPV42a and AtPV42b, respectively, which belong to the PV42 class of gamma subunits of SnRK1 complexes (Fang et al., 2011). SnRK1 complexes play a central role in regulating metabolic response and sugar signaling (Halford and Hardie, 1998; Halford et al., 2003; Lu et al., 2007; Polge and Thomas, 2007). AT2G28900 encodes *OUTER PLASTID ENVELOPE PROTEIN16-1* (*AtOEP16-1*). AtOEP16-1 plays in important role in photosynthesis by acting in NADPH:protochlorophyllide reductase A import into plastids (Samol et al., 2011). AT3G08940 encodes *LIGHT HARVESTING COMPLEX PHOTOSYSTEM II* (*LHCB4.2*). LHCB4.2 is a chlorophyll A/B binding protein that functions in photosynthesis (Boekema et al., 1999). AT4G09610 encodes *GAST1 PROTEIN HOMOLOG 2* (*GASA2*). GASA2 is postulated to act in response to gibberellins (Herzog et al., 1995). AT4G18650 encodes *DOG1-LIKE 4* (*DOGL4*). *DOGL4* is a member of a small gene family that includes *DOG1*, which functions in control of seed dormancy. However, the function of *DOGL4* is currently unknown (Bentsink et al., 2006). AT4G35770 encodes *ARABIDOPSIS THALIANA SENESCENCE 1/DARK INDUCIBLE 1* (*SEN1/DIN1*). SEN1/DIN1 is associated with senescence (Oh et al., 1996). AT5G07990 encodes *TRANSPARENT TESTA7* (*TT7*). TT7 is a flavonoid 3′ hydroxylase that acts in anthocyanin biosynthesis (Schoenbohm et al., 2000). AT5G10120 encodes *ETHYLENE INSENSITIVE 3-LIKE 4 PROTEIN* (*EIL4*). Computational analyses predict that EIL4 is a transcription factor (Riechmann et al., 2000) that might function in gibberellic acid signaling and/or in biosynthesis of gibberellins (Heyndrickx and Vandepoele, 2012). AT5G13930 encodes *CHALCONE SYNTHASE/TRANSPARENT TESTA4* (*CHS/TT4*). CHS/TT4 functions in anthocyanin biosynthesis (Feinbaum and Ausubel, 1988).

Gene expression was analyzed in seeds germinating in the presence of both 0.1 M Suc and 0.1 M sorbitol. Transcript levels of several of the genes are significantly induced by Suc in wild-type germinating seeds (Figure 6). None of the 10 genes analyzed was significantly Suc-repressed in wild-type germinating seeds. Exogenous Suc also significantly affected transcript levels for several of the genes in the *hac1* mutants.

## Discussion

The ability to sense and respond to the levels of available nutrients, such as Suc and Glc, is critical for all organisms. Although a number of forward genetic screens have been successful in identifying components of plant sugar-response networks (Zhou et al., 1998; Arenas-Huertero et al., 2000; Huijser et al., 2000; Laby et al., 2000; Gibson et al., 2001; Rook et al., 2001; Cheng et al., 2002; Rolland et al., 2002a; Huang et al., 2008, 2010), many components remain to be identified. A reverse genetic screen was thus conducted to identify additional components of sugar-response networks. The screen used in this work was based on the rationale that some of the genes involved in sugar-response networks are likely to be themselves sugar regulated at the steady-state mRNA level and to encode proteins with activities commonly associated with response networks.

This reverse genetic screen was successful in identifying mutant lines that exhibit increased sensitivity (AT5G51760, *AHG1*) or increased resistance (AT1G79000, *HAC1*) to the inhibitory effects of Suc and Glc on early seedling development. The identification of mutants defective in sugar response validates the rationale on which the reverse genetic screen was based. As *ahg1* mutants had previously been shown to have abscisic acid, Glc, Suc, and mannitol seed germination phenotypes (Nishimura et al., 2007), further experiments focused on *hac1* mutants. To test further whether mutations in *HAC1* are responsible for the sugar-insensitive phenotype, two additional *hac1* T-DNA insertion lines were characterized and found to exhibit increased resistance to the inhibitory effects of both Glc and Suc. These results indicate that mutations in *HAC1* are responsible for conferring the sugar-response phenotype. In addition, the finding that *hac1* and wild-type seedlings exhibit similar responses to concentrations of sorbitol that are equi-molar, or slightly greater than equi-molar, to the concentration of Glc and Suc used in the sugar-response assays indicates that the *hac1* mutants are not simply osmo-tolerant.

Quantitative RT-PCR was used to analyze *HAC1* transcript levels in *hac1-3, hac1-2, hac1-6*, and wild-type plants. The results of these experiments indicate that all three *hac1* alleles produce partial-length *HAC1* transcripts that are similar in abundance to the *HAC1* transcripts produced by wild-type plants. These *hac1* mutants might therefore produce partial HAC1 polypeptides. Whether the *hac1* mutants do produce partial HAC1 polypeptides and, if so, whether those polypeptides have any activity remains to be determined.

Although several forward genetic screens for Arabidopsis mutants that exhibit altered response to the inhibitory effects of Glc and/or Suc on early seedlings development have been performed (Zhou et al., 1998; Arenas-Huertero et al., 2000; Laby et al., 2000; Gibson et al., 2001; Cheng et al., 2002; Huang et al., 2008, 2010), there have been no reports of *hac1* mutants being identified during any of these screens. The reason that *hac1* mutants were not identified in these forward genetic screens may be because mutations in *HAC1* confer a relatively weak sugar-insensitive phenotype. For example, when germinated on media supplemented with 0.3 M Suc only ~25% of homozygous *hac1* seeds produce seedlings with true leaves. Thus, detection of the sugar-insensitive phenotype of individual *hac1* seeds during a forward genetic screen is problematic. In contrast, detecting the sugar-insensitive phenotype of groups of 50–100 *hac1* seeds during a reverse genetic screen is straightforward. It should also be noted that the fact that mutations in *HAC1* produce only a moderate sugar-insensitive phenotype does not demonstrate that histone acetyltransferase activity plays an unimportant role in sugar response. HAC1 is one of five related CBP/p300-like histone acetyltransferases in Arabidopsis (Pandey et al., 2002). Therefore, the possibility arises that HAC1 and one or more related histone acetyltransferases may play redundant, or partially redundant, roles in plant sugar response. Alternatively, the *hac1* mutants might produce truncated HAC1 polypeptides that might retain some HAC1 activity.

HAC1 has been shown to have histone acetyltransferase activity (Bordoli et al., 2001). Histone modification is a reversible method that cells use to control gene expression, with histone acetylation activating expression of specific sets of genes (reviewed in Lauria and Rossi, 2011). Histone modifications, including acetylation and deacetylation, are involved in controlling a wide variety of pathways ranging from hormone, stress, and light responses to mitosis, development, and Agrobacterium-mediated transformation (Xu et al., 2005; Chen et al., 2010; Desvoyes et al., 2010; Servet et al., 2010; Berr et al., 2011; Costas et al., 2011; Grafi, 2011; Magori and Citovsky, 2011; Qiao and Fan, 2011; Yaish et al., 2011; Luo et al., 2012). Of particular interest to this work are previous findings indicating a role for histone acetylation in sugar response. In mammals, Glc availability has been shown to affect histone acetylation (Wellen et al., 2009) and a p300 histone acetyltransferase has been shown to be involved in Glc-induced expression of a particular gene (Cha-Molstad et al., 2009). In *S. cerevisiae*, the histone acetyltransferase Gcn5 has been shown to interact with Snf1 (Lo et al., 2001), a protein kinase known to be vital to yeast Glc response pathways (Geladé et al., 2003). However, the Snf1-Gcn5 complex appears to be involved predominantly in inositol signaling (Lo et al., 2001).

Quantitative RT-PCR was used to identify some of the genes that have altered steady-state transcript levels in *hac1* mutants. These experiments resulted in the identification of nine genes that have significantly altered transcript levels in *hac1* mutants. Expression of eight of these genes is lower in *hac1* than in wild-type, suggesting that HAC1 functions as a positive regulator of these genes. As histone acetylation is known to increase transcription rates of associated genes, these results are consistent with HAC1 playing a direct role in the regulation of these genes. However, the possibility that HAC1 affects expression of these genes indirectly cannot be ruled out at this time. HAC1 may regulate these genes, directly or indirectly, by binding to transcription factors or other histone modifiers in order to provide more specific regulation via multiple histone modifications taking place simultaneously. Alternatively, HAC1 could act by providing a scaffold for multiple transcription factors to bind to in order to activate target genes (Bharti et al., 2004). Or, HAC1 might simply be acting as a histone acetyltransferase in control of gene expression. Transcript levels of AT3G08940 (*LHCB4.2*) are higher in *hac1* than in wild-type germinating seeds. This result suggests that HAC1 acts indirectly as a negative regulator of *LHCB4.2* expression.

The expression levels of five genes that were shown previously to be sugar regulated were examined in *hac1* and wild-type germinating seeds. AT2G28900 (*AtOEP16-1*) and AT3G08940 (*LHCB4.2*) encode proteins important in photosynthesis (Boekema et al., 1999; Samol et al., 2011). Expression of *AtOEP16-1* is increased by Suc (Gonzali et al., 2006) and is also regulated in response to Glc (Price et al., 2004). Expression of *LHCB4.2* is repressed by Suc (Rogers et al., 2005). In the experiments performed as part of this work, *AtOEP16-1* transcript levels were significantly Suc-induced in wild-type germinating seeds. However, Suc had no statistically significant effects on expression of *LHCB4.2* in wild-type germinating seeds. This apparent discrepancy between the results presented here and previous results may be due to the fact that germinating seeds were analyzed in the experiments reported here. AT5G07990 (*TT7*) and AT5G13930 (*CHS/TT4*) play important roles in anthocyanin biosynthesis (Feinbaum and Ausubel, 1988; Schoenbohm et al., 2000). *TT7* (Solfanelli et al., 2006) and *CHS/TT4* (Tsukaya et al., 1991; Solfanelli et al., 2006) transcript levels are induced by Suc. In the experiments performed as part of this work, Suc increased expression of *TT7* and *CHS/TT4* in wild-type germinating seeds. Expression of AT4G35770 (*SEN1/DIN1*) is repressed by Suc and Glc but not by mannitol or 3-O-methyl-D-glucose (Chung et al., 1997). Expression of *SEN1/DIN1* was not found to be consistently regulated by Suc in wild-type germinating seeds in this study. This apparent discrepancy may be due to the fact that germinating seeds were analyzed in this study.

Transcript levels of AT1G15330 (*AtPV42a*), AT1G80090 (*AtPV42b*), AT4G09610 (*GASA2*), and AT4G18650 (*DOGL4*) were found as part of this work to be Suc induced in wild-type germinating seeds. However, these effects were typically modest. Transcript levels of several of the above genes are also Suc regulated in the *hac1* mutants. Although some differences were observed between Suc regulation of these genes in *hac1* vs. wild-type germinating seeds, no convincing evidence of a consistent effect of the *hac1* mutants on Suc-regulated gene expression was obtained. This result might be due to the relatively modest effects of Suc on gene regulation in wild-type germinating seeds that were observed during this study. Alternatively, HAC1 might not affect Suc regulated gene activity of the genes assayed at the steady-state mRNA level.

Results reported here that *hac1* mutants are resistant to the inhibitory effects of high concentrations of Glc and Suc on early seedling development indicate that HAC1 plays a role in plant sugar response. Involvement of a histone acetyltransferase in sugar response provides a possible mechanism whereby nutritional status could have long-term effects on plant development and expression of specific genes. Results showing that mutations in *HAC1* lead to decreased expression of both AtPV42a and AtPV42b suggest a possible mechanism via which HAC1 may affect plant sugar response. AtPV42a and AtPV42b are the sole Arabidopsis members of the PV42 gene family (Fang et al., 2011). PV42 proteins represent one of three families of plant proteins that exhibit significant sequence similarity to the yeast SNF4 protein. SNF4 acts as a regulatory subunit of yeast SNF1 protein complexes (Celenza et al., 1989). SNF1 protein complexes are turned off by high levels of Glc and are required for the derepression of Glc-repressed genes (reviewed in Ghillebert et al., 2011). Plant SnRK1 complexes are homologous to yeast SNF1 complexes (reviewed in Ghillebert et al., 2011). Plant SnRK1 complexes function as central regulators of nutrient and energy status (Halford and Hardie, 1998; Halford et al., 2003; Baena-González et al., 2007; Lu et al., 2007; Polge and Thomas, 2007; Halford and Hey, 2009; Jossier et al., 2009; Radchuk et al., 2010; Delatte et al., 2011). In addition, mutations in *PRL1* cause increased SnRK1 activity and increased sensitivity to the inhibitory effects of high sugar concentrations on early seedling development (Németh et al., 1998; Bhalerao et al., 1999). Thus, increased SnRK1 activity has been associated with increased sugar sensitivity. Mutations that decrease SnRK1 activity may therefore cause decreased sugar sensitivity. By decreasing expression of AtPV42a and AtPV42b, mutations in *HAC1* may be decreasing SnRK1 activity and, consequently, sugar sensitivity.

The *hac1* mutants exhibit several other phenotypes in addition to sugar response. For example, mutations in *HAC1* have been reported previously to cause delayed flowering times in Arabidopsis by indirectly increasing the expression of the central floral repressor, FLC (Deng et al., 2007; Han et al., 2007). Interestingly, time to flowering has also been suggested to be partially regulated by sugar levels (Bernier et al., 1993; Corbesier et al., 1998; Roldán et al., 1999; van Dijken et al., 2004; Funck et al., 2012; Wahl et al., 2013). Thus, the results reported here and previously reported results raise the possibility that HAC1 might play a role in linking sugar response and time to flowering.

Mutations in *HAC1* also cause moderate resistance to the inhibitory effects of paclobutrazol on seed germination. Paclobutrazol inhibits seed germination by inhibiting biosynthesis of gibberellins. Other sugar-response mutants have also been found to exhibit paclobutrazol-resistant phenotypes (Laby et al., 2000; Gibson et al., 2001; Huang et al., 2008). The paclobutrazol-resistant seed germination phenotype of *hac1* mutants might be due, in part, to reduced expression of *GASA2* and *EIL4* in *hac1* mutants. GASA2 is postulated to act in response to gibberellins and *GASA2* transcripts are most abundant in siliques and dry seeds (Herzog et al., 1995). Computational analyses of the predicted amino acid sequence of EIL4 suggest that EIL4 is a transcription factor (Riechmann et al., 2000) that might function in gibberellic acid signaling and/or in biosynthesis of gibberellins (Heyndrickx and Vandepoele, 2012).

Previous results (Deng et al., 2007; Han et al., 2007) and results presented here indicate that *hac1* mutants have decreased fertility. In particular, *hac1* mutants produce short siliques that contain few seeds. Reduced expression of AtPV42a and AtPV42b has been shown previously to cause shorter siliques and reduced seed set in Arabidopsis. This decrease in fertility was shown to be the result of defects in stamen development and attraction of pollen tubes to female gametophytes (Fang et al., 2011). Thus, the decreased expression of AtPV42a and AtPV42b in *hac1* mutants may play a role in both the sugar-insensitive and reduced fertility phenotypes displayed by these mutants.

## Materials and methods

### Plant material, growth conditions, and media

Seeds from the *hac1-2* (SALK_070277), *hac1-3* (SALK_080380), *hac1-6* (SALK_122894), and Col-0 wild-type (CS60000) lines were obtained from the Arabidopsis Biological Resource Center (ABRC). The mutant seeds obtained from the ABRC were segregating for their respective mutations in *HAC1*. PCR was used to identify individual plants that were homozygous for each mutation in *HAC1*. Seeds of *spy-3* (Jacobsen and Olszewski, 1993), which is also in the Col-0 background, were obtained from Dr. Neil Olszewski. Unless otherwise noted, seeds were sown directly onto soil (Sunshine Mix LB-3 or BM2) and grown to maturity under continuous fluorescent light at approximately 22°C. Minimal Arabidopsis media was prepared as indicated (Kranz and Kirchheim, 1987).

### Sugar and paclobutrazol response assays

For each assay, 50–100 seeds were surface sterilized, stratified in the dark at 4°C for 3 days and then sown on Petri plates containing solid minimal Arabidopsis media (Kranz and Kirchheim, 1987) supplemented with the indicated additive(s). For sugar-response assays, the Petri plates were incubated under continuous fluorescent light at 22°C for 2 weeks prior to scoring for seed germination, cotyledon expansion, and true leaf formation, as described (Laby et al., 2000). For paclobutrazol-response assays, the Petri plates were incubated under continuous fluorescent light at 22°C for the indicated periods of time prior to scoring percent seed germination. Seed germination is defined as the emergence of any part of the seedling from the seed coat.

### Seed and silique number assays

Plants to be analyzed were grown to maturity in separate pots. The average number of siliques per plant was determined by counting all of the siliques produced by individual plants. The average number of seeds per silique was determined by counting the number of seeds in each silique produced by individual plants, or in every other silique produced by individual plants. The average number of seeds produced per plant was determined by multiplying the number of seeds produced per silique times the number of siliques produced per plant.

### RNA isolation, genechip and quantitative RT-PCR experiments

To obtain tissue for RNA isolation, seeds were surface sterilized and imbibed at 4°C in the dark for 3 days. Seeds were then sown on nytex screens on Petri plates containing minimal Arabidopsis media (Kranz and Kirchheim, 1987) and placed in a growth chamber under continuous fluorescent light at 22°C. After 20 h, the nytex screens with the seeds were transferred to Petri plates containing minimal Arabidopsis media supplemented with the indicated additives. The Petri plates were then incubated under the same conditions for an additional 12–13 h. Seed tissue was pooled from groups of three to four Petri plates and stored at −80°C. Seeds were then crushed using dry ice and a mortar and pestle. RNA was extracted from ~0.1 g aliquots of powdered seed tissue using Sigma-Aldrich's Spectrum Plant Total RNA kit with on column DNase1 treatment, according to the manufacturer's instructions (Sigma, St. Louis, MO). Preliminary GeneChip analyses using Affymetrix ATH1 GeneChips were performed by the University of Texas Medical Branch Molecular Genomics Core facility using ~30 μg aliquots of total RNA. Data were analyzed using the Expressionist software suite. For quantitative RT-PCR analyses, first strand cDNA was synthesized using the Promega Improm-II Reverse Transcription System (Promega, Madison, WI). Quantitative PCR was performed with an Applied Biosystems 7500 Real-Time PCR Machine using the SYBR Green JumpStart Taq ReadyMix (Sigma, St. Louis, MO). Primer pair sequences are listed in Table 1. Data were normalized using the geometric mean (Vandesompele et al., 2002) of the results obtained for two control genes, the Arabidopsis *ACT7* and *UBQ6* genes.

**Table 1:** **Primer sets used for qPCR**.

**Name**	**Forward primer (5′-3′)**	**Reverse primer (5′-3′)**
ACT7	TGCTGACCGTATGAGCAAAG	GATTGATCCTCCGATCCAGA
AT1G15330	TAGTGCCAGTGGAGAGCAGC	TCAGGTGCATGAACGATGGGGAC
AT1G80090	TGCTCAATGCGGTTCCTATC	AGCCGAGAATGTCCCCACC
AT2G28900	CAATCTCACCGTTGATGCCT	CGCATGCTCCAAAGTGCTC
AT3G08940	GAGCTGATTCACGGTCGGTG	AGCTCTACCTTGCCGGCGT
AT4G09610	ACTTATGAGCTTCACGTCCACGCT	ACAGCTGTTGCACGCTCTCAAGC
AT4G18650	TTATACAGCCAAATGGGCAGC	CGAAACACCATCGACGGTT
AT4G35770	GGAAAGCAACGACAACAAGCA	CCTCACGTCGAGATAGCGGT
AT5G07990	GACACGTCAGCAAGTACGGTG	CGGCCCACAACAATATCAAG
AT5G10120	TTCACGGCGCAAGAAGATG	TGGTTTACCCTTTTCCGGTACA
AT5G13930	GGATCTCGCCGAGAACAATC	GGGAGTCAAGGTGGGTGTCA
HAC1 pair 1	TGGATACCAACATTCATCGAG	TTCTCTGGGAGCTCATGGAT
HAC1 pair 2	TGGCAATCTCAGTCACAGGA	CTTCATCCGTTCCAGACATTC
HAC1 pair 3	ATATCCGTGGTCTCCGTCAG	CTGCACATAGTTGGCAGGAA
HAC1 pair 4	GCAATTGCGAGTCTTGCAGC	TCACTTTCCGGCAATTCGG
UBQ6	CCATCGACAATGTCAAGGCC	GGTACGTCCGTCTTCGAGCT

### Conflict of interest statement

The authors declare that the research was conducted in the absence of any commercial or financial relationships that could be construed as a potential conflict of interest.
